# The Use of Piperidinium Surfactants in Nematicide Formulations

**DOI:** 10.3390/molecules31091470

**Published:** 2026-04-29

**Authors:** Rushana Kushnazarova, Alla Mirgorodskaya, Eugeny Nikitin, Anastasia Egorova, Alsu Gatiyatullina, Tatiana Kalinnikova, Lucia Zakharova

**Affiliations:** 1 Arbuzov Institute of Organic and Physical Chemistry, FRC Kazan Scientific Center of RAS, Arbuzov Str. 8, 420088 Kazan, Russiaberkutru@mail.ru (E.N.); luciaz@mail.ru (L.Z.); 2 Research Institute for Problems of Ecology and Mineral Wealth Use of Tatarstan Academy of Sciences, Daurskaya Str. 28, 420087 Kazan, Russiagaf9212@gmail.com (A.G.); tbkalinnikova@gmail.com (T.K.)

**Keywords:** piperidinium surfactants, nematodes, carbofuran, synergistic effect, lipid membrane

## Abstract

A series of hexadecylpiperidinium surfactants containing alkyl (PMe-16, PEt-16, PBu-16), benzyl (Benz-16, 1-Benz-3-HP-16, 1-Benz-4-HP-16), and hydroxyl (3-HPMe-16, 4-HPMe-16) substituents in the ring were tested with the nematode *Caenorhabditis elegans* to investigate the relationship between nematocidal activity and the structural features of surfactants. It was found that increasing the hydrophobicity of the substituent in the surfactant head group reduced the nematocidal activity in the order PMe-16 > PEt-16 > PBu-16 > Benz-16. The lead compound, PMe-16, showed significantly higher activity than the commercial insecticide carbofuran, and was able to induce nearly complete nematode mortality within 24 h at a concentration of 50 μg·mL^−1^, as well as suppress culture development at concentrations of 25–100 μg·mL^−1^. All tested piperidinium surfactants inhibited nematode population development at 100 μg·mL^−1^, while PMe-16 remained effective at concentrations as low as 25 μg·mL^−1^. The membranotropic properties of the surfactants were evaluated using a turbidimetric method with dipalmitoylphosphatidylcholine (DPPC)-based liposomes as a model of biomembranes. Dynamic light scattering measurements were performed in parallel to assess changes in liposome size and zeta potential as a function of surfactant content, as well as to determine the critical concentration required to induce lipid bilayer destabilization. These results provide indirect evidence of surfactant–membrane interactions. The combinations of piperidinium surfactants and carbofuran showed pronounced synergistic effects, reducing the insecticide dose while maintaining efficacy. Synergy was evaluated using the Bliss independence model and the Highest Single Agent model. The addition of the most active surfactants (PMe-16 and 4-HPMe-16) at 6.25 μg·mL^−1^ enabled an approximately twofold reduction in the carbofuran dose while maintaining full nematocidal activity.

## 1. Introduction

The amphiphilic nature of surfactant molecules explains their numerous unique properties and wide applications in various fields of science and industry. Their ability to integrate into lipid membranes underlies their antimicrobial, antitumor, and antioxidant effects [1,2,3]. The adsorption of surfactants at phase interfaces allows the control of surface wettability [4,5,6]. Surfactants act as nanocarriers for biologically active compounds in their aggregated forms, such as micelles and vesicles, enhancing their solubility, improving bioavailability, facilitating membrane penetration, and enabling targeted delivery [7,8,9,10].

These properties form the foundation for the use of surfactants in agriculture as adjuvants in pesticide formulations. Adjuvants can improve the effectiveness of pesticide treatments by increasing the plant uptake of active ingredients. They also ensure uniform coverage and help to retain these compounds on the treated surfaces [11,12]. Surfactants, which combine surface activity with inherent antimicrobial effects against plant pathogens, can be considered as multifunctional adjuvants, expanding the range of beneficial properties offered by pesticide formulations [13,14]. The biological activity of surfactants depends on several key structural parameters, such as the nature and charge of the polar head group and the hydrophobic tail length. While nonionic amphiphiles are often preferred in agrochemical formulations due to their low toxicity and good environmental compatibility [11,15,16], cationic surfactants sometimes exhibit superior biological activity. These cationic compounds have a strong affinity for cell membranes and an enhanced ability to penetrate biological barriers due to electrostatic interactions with negatively charged biological surfaces [17,18,19].

Among the various cationic surfactants, piperidinium derivatives have gained attention due to their high antimicrobial and fungicidal properties. Our previous studies have shown that hexadecylpiperidinium surfactants, which differ in the substituents on the heterocyclic head group, exhibit strong activity against a wide range of Gram-positive and Gram-negative bacteria, including antibiotic-resistant strains, as well as pathogenic fungi [19,20,21,22]. In some cases, their effectiveness matched or even exceeded that of commercial antibiotics. Furthermore, combinations of these surfactants with commercially available fungicides, such as tebuconazole, have demonstrated significant synergistic antimicrobial effects [13].

In this work, the effects of piperidinium surfactants on nematodes, as well as their potential to act as adjuvants enhancing the effectiveness of commercial nematicides, were evaluated. Nematodes are microscopic soil-dwelling parasites that feed on plant cells, causing extensive damage to roots, stems, leaves, and fruits, leading to reduced yield, poor product quality, and, in severe cases, the death of plants [23,24,25]. They are a major concern for agriculture and have a significant economic impact.

Thorough analysis of the literature revealed that no systematic studies are available on the application of surfactants in the research on nematode management. At present, two different directions can be differentiated in investigations of the surfactant-based systems. The first direction covers the use of surfactants as adjuvants to increase the effectiveness of nematicides [26,27,28,29,30]. This can be exemplified by the fabrication of nanoemulsion formulations of essential oils stabilized by nonionic surfactants Span 20, Tween 80 and Triton-X-100 [26]. In this study, the nematocidal effect of different active natural ingredients (garlic, citronella and *Annona* oils) was compared, with no attention paid of the surfactant role. In [27], oil-in-water emulsions based on plant extracts possessing nematocidal effects were developed with the use of different types of surfactants, including calcium dodecylbenzene sulfonate, Span 80, and Tween 80. Similarly, no investigations on the factors that mediated the surfactant activity or the effect of surfactant structure were discussed. Different pesticide formulations involving nonionic surfactant Tween 80, amphoteric surfactant dodecyl dimethyl betaine, cationic surfactant hexadecyl trimethyl ammonium bromide, and anionic surfactant sodium 2-butyl-1-naphthalenesulfonate were proposed in [29]. Although the surfactant structure was varied, this was not monitored from the viewpoint of changes in biological effect. Moreover, this study focused on the general pesticide effect, rather than on specific nematocidal activity. Interestingly, formulations of entomopathogenic nematodes were used against the tomato leaf miner [30]. While surfactant-based nanocompositions demonstrated one of the highest protective effects, their formula was denoted as undisclosed. It should be emphasized that these segmentary publications are conducted under varying experimental conditions, which makes it difficult to directly compare the reported results. Another line of research on the effects of surfactants on nematodes is devoted to the safety testing of different classes of amphiphilic compounds using model organisms. Although this field only has an indirect relation to our study, it also provides evidence that the specific surfactant nature was beyond these investigations [31,32,33].

The above analysis clearly demonstrates that the publications available provide limited insight into the structural factors governing surfactant–nematode interactions and the key properties underlying their function as adjuvants. At the same time, these studies strongly support high efficacy of the surfactant-based formulations, which encourages researchers to enhance their activity in this field. Importantly, even beyond the specific nematode topic, the majority of investigations focusing on surfactant biological activity emphasize the role of their hydrophobicity, i.e., the effect of alkyl chain length, while the role of head group structure is insufficiently discussed. Essentially, the present paper is one of pioneer work highlighting the role of surfactant structural behavior in the effectiveness of nematocidal formulations.

**Figure 1:**
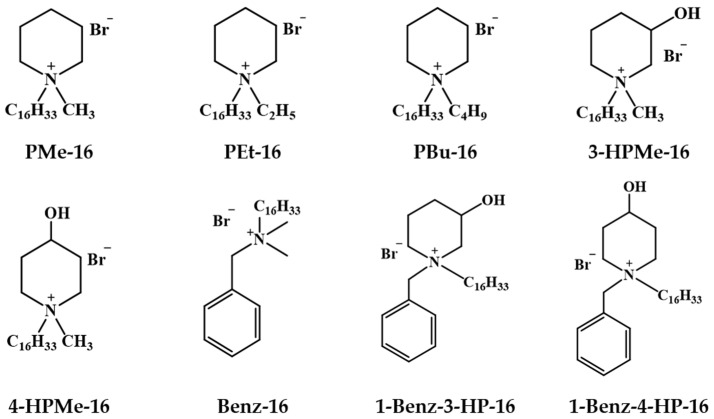
Structural formulas of the hexadecylpiperidinium surfactants.

In this regard, the current study aimed to investigate the effects of the hexadecylpiperidinium surfactants containing alkyl, hydroxyl, and benzyl substituents in the heterocyclic head group on nematode activity (Figure 1). *Caenorhabditis elegans* was selected as a representative model organism for experimental evaluation, and carbofuran (CBF) was used as a reference insecticide. CBF is a carbamate pesticide widely used in agriculture and is characterized by well-documented biological activity [34,35], providing a reliable benchmark for comparison with the responses observed for the investigated surfactant systems. In addition, the potential of cationic surfactants as adjuvants was assessed using representative acetylcholinesterase-inhibiting insecticides, including CBF and, in selected experiments, aldicarb. This approach was used to determine whether these surfactants could enhance the activity of the active compounds, thereby influencing their effective application levels.

## 2. Results and Discussion

### 2.1. Nematocidal Activity of the Piperidinium Surfactants

The free-living soil nematode *C. elegans* is a convenient model organism for studying neurotoxicology and ecotoxicology. It has several advantages over other model systems, including ease and low-cost laboratory cultivation, safety for researchers, rapid generation time, and high reproductive capacity [36,37,38,39]. This organism is widely used in experimental research due to its well-characterized body plan, physiology, and neurochemistry, which share significant similarities with plant-parasitic and veterinary-important nematodes [37,38,40,41,42]. Its ease of culture, short life cycle, and well-understood genetic and physiological responses to chemical agents make it an ideal model for the preliminary screening of nematocidal activity. Although *C. elegans* is not a plant-pathogenic species, several studies have demonstrated that the results obtained using this model can be biologically relevant for phytopathogens. For example, the toxicity of fungal culture filtrates against *C. elegans* correlates with the activity against the root-knot nematode *Meloidogyne incognita*, which reduces tomato root damage in vitro [43]. This supports the usefulness of *C. elegans* in identifying potentially active compounds against plant-parasitic nematodes. Furthermore, a recent review highlights that the mechanisms of action of toxic compounds and detoxification pathways in *C. elegans* share key similarities with those in plant-parasitic nematodes, supporting its use for initial structure–activity relationship analysis prior to validation in target species [44].

**Figure 2:**
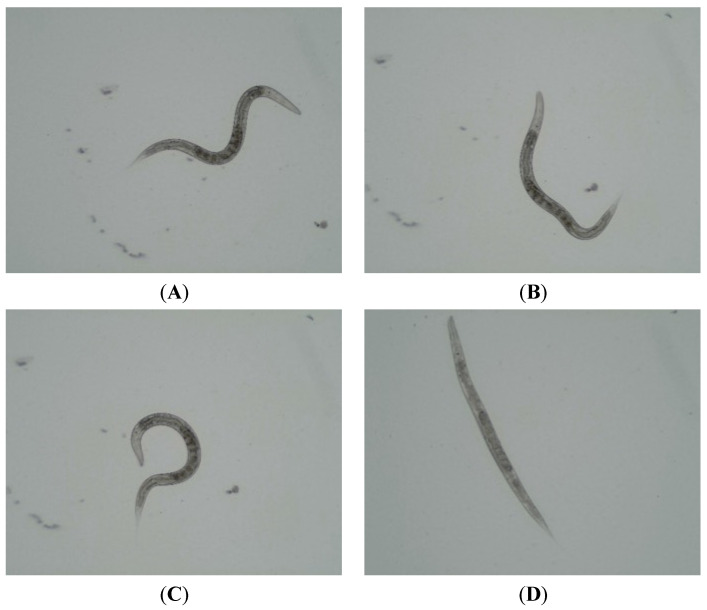
Microphotographs of nematodes at different experimental stages: (**A**) live nematode exhibiting normal locomotion; (**B**,**C**) live nematodes showing impaired locomotion; (**D**) dead nematode.

The nematocidal activity of piperidinium surfactants was evaluated using a serial dilution method over a concentration range of 100.0–6.5 µg·mL^−1^ with young adult *C. elegans* of the wild-type N2 strain. Table 1 summarizes the experimental data on nematode mortality within 24 h. To visually facilitate a comparison of the results, images of nematodes at different experimental stages were obtained using an MSHOT MF23 fluorescence microscope at 10× magnification (Figure 2).

The half-maximal effective concentration (EC_50_), estimated by the Behrens method [45], is provided in the Supplementary Materials (SM) (Figure S1). The results show that the hydrophobicity of the substituents in the piperidinium head group influences the surfactant’s effect. Activity decreased in the following order: PMe-16 > PEt-16 > PBu-16 > Benz-16. Surfactants containing the benzyl fragment caused no more than 30% mortality, even at the highest tested concentrations. The introduction of hydroxyl groups did not significantly enhance activity, although the para-substituted derivative (4-HPMe-16) exhibited slightly higher potency than the meta-substituted analogue (3-HPMe-16). Notably, the most active compounds markedly outperformed the commercial insecticide CBF (Table 1) and achieved near-complete nematode mortality at 50 μg·mL^−1^ within 24 h.

In addition to the effects on adult nematodes, piperidinium surfactants were also found to affect the development of nematode populations under the studied conditions. Table 2 summarizes the effects of the most active compounds on the lysis of nematode eggs. At a concentration of 100 μg·mL^−1^, all tested surfactants inhibited the development of the culture, while PMe-16 remained effective even at 25 μg·mL^−1^. It should be noted that this assessment is based on qualitative observations of culture development over time and does not include quantitative measures of egg viability. However, the 10-day observation period covers multiple reproductive cycles of *C. elegans*, allowing for the reliable detection of both developmental delays and complete suppression of population growth.

It should be noted that the concentrations of the surfactants used in this study were below their critical micelle concentration (CMC). The CMC for these compounds ranged from 0.3 to 1.0 mM, or 200–400 μg·mL^−1^ [20,21]. This suggests that the surfactants are active in their monomeric (non-aggregated) form. Based on their amphiphilic nature, these compounds are expected to interact with and insert into lipid bilayers, leading to membrane loosening and partial disruption of lipid packing. These changes may increase membrane permeability and potentially contribute to the observed biological effects [46,47,48]. This interpretation provides a working hypothesis that can be tested using model lipid systems in order to assess the membrane-active properties of piperidinium surfactants.

### 2.2. Membranotropic Properties of the Piperidinium Surfactants

The nematode’s cuticle and surface coat form the outermost protective layer, serving as a primary barrier against environmental stressors and biological threats, such as pathogens and predatory organisms [49,50]. This structure is composed of cross-linked collagens, glycoproteins, and lipids that are produced and secreted by the hypodermal cells [50]. In *C. elegans,* the cuticle surface is characterized by a high lipid content, exceeding 81% of the surface composition [49]. This emphasizes the critical role of lipids in maintaining structural stability and regulating permeability. The membranotropic activity of hexadecylpiperidinium surfactants was evaluated using DPPC-based liposomes, which are commonly used as simple biological membrane models. The properties of phospholipid membranes are largely determined by the main phase transition temperature (T_m_), which characterizes the structural change of a lipid from the ordered gel phase to the disordered liquid-crystalline phase. A decrease in T_m_ indicates a perturbation of the lipid bilayer, for example, due to the incorporation of foreign molecules [51]. The effect of surfactants on T_m_ was monitored via turbidimetric titration. For a single DPPC, this transition occurs at 41 ± 1 °C [51,52].

**Figure 3:**
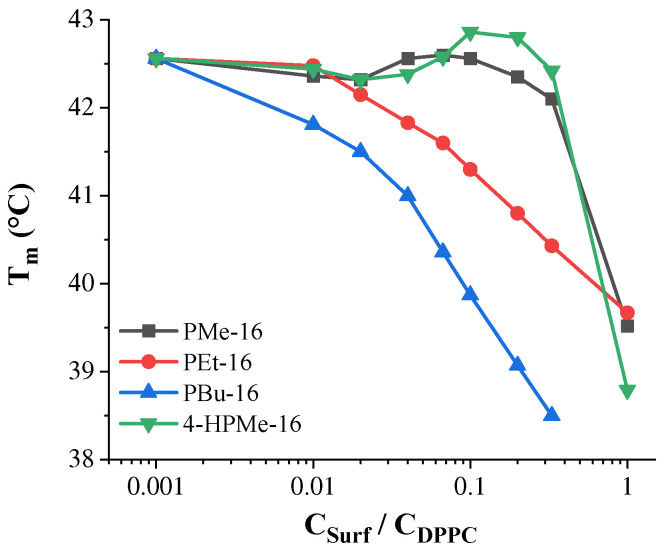
DPPC phase transition temperature vs. piperidinium surfactant/lipid molar ratio.

Figure 3 illustrates the dependence of the T_m_ of DPPC on the molar ratio of piperidinium surfactants-to-lipid, as determined from turbidimetric curves (Figure S4). The surfactants integrated into the lipid bilayer at low concentrations, causing only a slight decrease in T_m_. The methyl-substituted derivatives (PMe-16 and 4-HPMe-16) exhibited minimal changes up to a surfactant-to-lipid molar ratio of 1:3, with T_m_ values of 42.05 °C and 42.42 °C, respectively (Table 3, Figure 3). These results suggest that the surfactants intercalate into the bilayer without significantly disrupting lipid packing. In contrast, surfactants with longer alkyl substituents in the piperidinium head group, such as PEt-16 and PBu-16, caused more pronounced reductions in T_m_ even at lower surfactant-to-lipid ratios (Table 3). At a 1:5 molar ratio, T_m_ decreased to 40.80 °C for PEt-16 and 39.07 °C for PBu-16. At higher ratios, T_m_ continued to decrease, reaching 39.67 °C for PEt-16 and 38.50 °C for PBu-16 at the 1:1 and 1:3 molar ratios, respectively. Significant bilayer destabilization was observed at surfactant-to-lipid ratios approaching equimolar for all systems tested (Table 3). These results are consistent with previous reports showing that the incorporation of hydrophobic cationic amphiphiles into lipid bilayers progressively lowers T_m_ and perturbs lipid packing, leading to destabilization of the bilayer structure.

Dynamic light scattering measurements were carried out in parallel with turbidimetry to evaluate changes in the size of liposomes and zeta potential (ZP). DPPC liposomes exhibited a hydrodynamic diameter of 106 nm and a narrow size distribution with a polydispersity index (PdI) of 0.05, as well as a ZP of 4.1 ± 0.2 mV (see Table 3). Upon the introduction of surfactants, the particle size and surface charge both increased, as well as the PdI value (Table 3). However, at surfactant-to-lipid ratios corresponding to the critical region of the T_m_ decrease (typically near equimolar), particle sizes could not be accurately determined in some cases (Figures S5–S12). This reflects the structural destabilization of the lipid bilayer.

It should be noted that the surfactant concentrations needed to induce bilayer destabilization were approximately three times higher than those that caused 100% nematode mortality for PMe-16, PEt-16, and 4-HPMe-16 (Table 1 and Table 3). In the case of the butyl derivative (PBu-16), this effect was observed at an approximate 1:1 surfactant-to-lipid molar ratio. These results suggest that the biological activity observed in nematode assays cannot be explained solely by complete membrane disruption in model lipid systems. Rather, it may involve more subtle alterations in membrane organization and physicochemical properties. However, these conclusions are based on simplified lipid model systems, and therefore should be interpreted with caution when attempting to apply them to biological systems.

Additional mechanisms that may influence the permeability of the cuticle are likely to contribute to the observed activity. However, their exact role at this stage remains hypothetical. In particular, interactions between surfactants and the collagen matrix of the *C. elegans* cuticle could potentially play a role. It has been reported that ionic surfactants can directly interact with collagen and affect its structural and rheological properties. For example, charged surfactants such as hexadecyltrimethylammonium bromide have been shown to alter the thermal stability, conformation, and viscosity of collagen solutions [53,54]. Moreover, studies on collagen–surfactant systems indicate that cationic surfactants significantly affect the surface activity and mechanical properties of collagen layers [55] while also altering the hydration and dynamics of collagen macromolecules [56].

Thus, the current findings suggest that changes in lipid packing may contribute to the observed activity of the piperidinium surfactants on nematodes, rather than complete membrane disruption. However, additional mechanisms cannot be ruled out and require further investigation.

### 2.3. Piperidinium Surfactants as Adjuvants in Nematocidal Compositions

Surfactants are widely used as agricultural adjuvants to improve the effectiveness of pesticide formulations [13,57,58,59]. At low concentrations, they can enhance pesticide efficacy by improving the interaction of active substances with their biological targets, thereby enabling a reduction in the applied dose and decreasing the overall chemical load on agroecosystems, which is particularly important for the rational use of crop protection agents. Piperidinium surfactants were used as adjuvants in nematocidal formulations in combination with CBF, given that these surfactants exhibit nematocidal activity. Their combined use was considered beneficial for enhancing the efficacy and reducing the effective dose of insecticide. CBF is highly toxic to warm-blooded organisms [60,61,62]. However, it continues to be an effective insecticide and nematocide for controlling a variety of agricultural pests. In addition, seed treatments with CBF-based formulations offer long-term protection against soil-borne pests [34,35]. Consequently, reducing the amount of CBF used by adding adjuvants can lower the toxicity of insecticide formulations and reduce their overall environmental impact.

**Figure 4:**
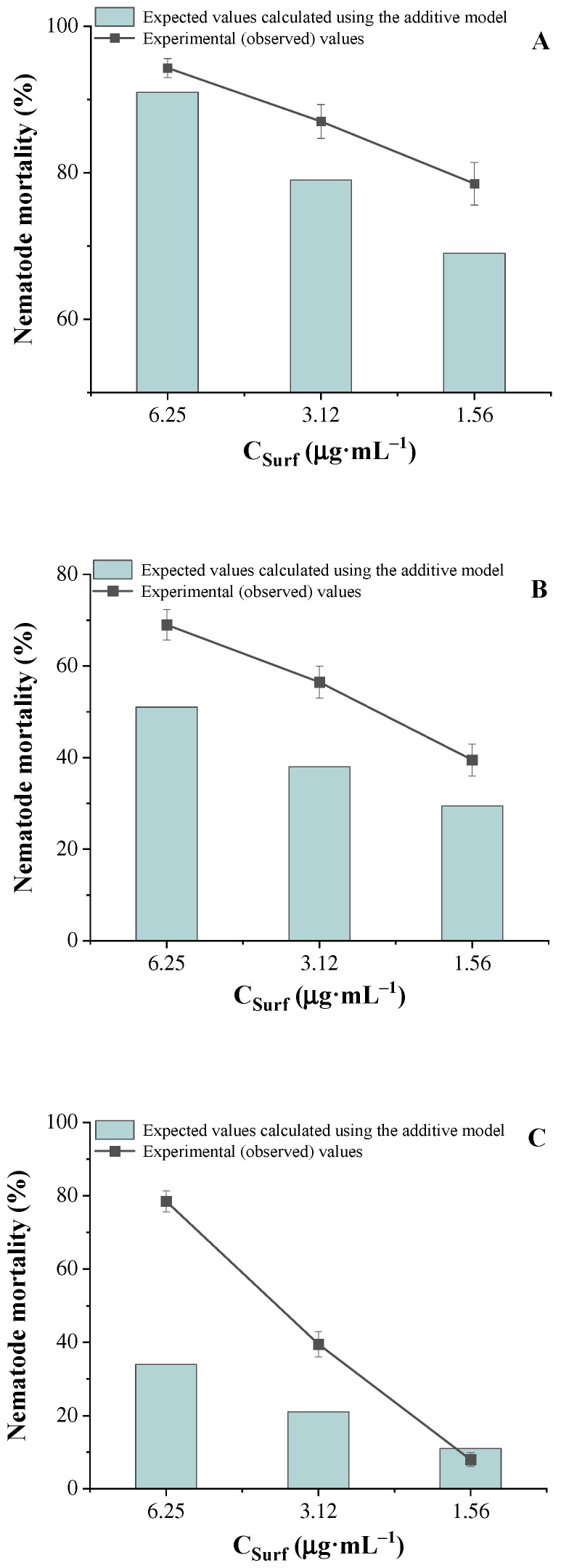
Mortality of nematodes exposed to PMe-16/CBF formulations at different component concentrations. Panels (**A**–**C**) correspond to CBF concentrations of 100, 50, and 25 µg·mL^−1^, respectively. The data are presented both as experimentally observed values and as those calculated using the additive model.

The results of nematocidal assays for binary mixtures containing various ratios of CBF and piperidinium surfactants are presented in Table 4 and Figure 4. These data represent the experimentally observed nematocidal activity at different concentrations of the components. In all tested systems, an increase in nematode mortality was observed in the mixtures compared to the individual components, indicating an enhanced combined effect.

To determine whether this enhancement can be attributed to simple additivity or interaction between the components, models of combined action were used. Various approaches for evaluating combination effects have been widely used, primarily in pharmacology and medicine [63]. They can be correctly extrapolated in other applications, e.g., pesticide formulations. In particular, the Bliss independence model, based on the principle of the independent action of components, was used as a reference additive model [64,65,66]. According to this model, when two agents act independently, the joint survival probability (*S*_AB_) equals the product of the survival probabilities obtained for each agent when applied alone [67]:

(1) $$S_{AB}=\;S_{A}\times S_{B},$$

where *S*_A_ and *S*_B_ are the fractions of surviving organisms after exposure to agents A and B, respectively.

Since mortality is related to survival as M=1−S, the expected mortality for independent action can be expressed as:

(2) $$M_{AB}=\;M_{A}+M_{B}\;-\;M_{A}\times M_{B}$$

where *M*_A_ and *M*_B_ are the observed mortalities (in fractions from 0 to 1) for each component applied separately.

The expected additive values were calculated using Equation (2), and the detailed results are summarized in Table S1. Comparison of the experimental data with the predicted additive values revealed a significant synergistic effect in all tested systems. In each case, the observed nematocidal activity was greater than the additive predictions based on the Bliss model. The most pronounced synergy was observed for the PMe-16/CBF system at a concentration of 25.0 μg·mL^−1^ of CBF and 6.25 μg·mL^−1^ surfactant, where the interaction coefficient was 2.47 (see Figure 4 and Table S1). However, this coefficient should be regarded as a comparative descriptor rather than a precise quantitative measure of interaction strength.

To further validate these findings, the Highest Single Agent (HSA) model, also known as Gaddum’s noninteraction or cooperative effect [63,68], was applied as an independent reference approach. This method does not require the approximation of dose–response relationships and provides independent confirmation of the enhanced effect observed in the Bliss model. The HSA model evaluates whether the observed effect is significant in comparison to the most effective individual agent, without accounting for the expected additive effect of both agents in combination [69]. Using this approach, the combination index (CI) was calculated as follows: CI = max(*M*_A_,*M*_B_)/*M*_A,B_. The CI values (<1 for almost all investigated systems) indicate the presence of synergistic interactions (a positive combined effect), which are consistent with the conclusions drawn from the Bliss model (Table S2). To visually represent the data for the HSA model, graphs of mixed systems containing 6.25 μg·mL^−1^ of piperidinium surfactants and 100 μg·mL^−1^ of CBF are presented in the SM (Figure S14). The agreement between the Bliss- and HSA-based analyses indicates a consistent enhancement of the observed effect in the presence of surfactants while also suggesting a high level of agreement between the different evaluation approaches; however, the magnitude of the inferred interaction varies depending on the reference model applied.

Importantly, the addition of cationic piperidinium surfactants at a concentration of 6.25 μg·mL^−1^ allowed the CBF dose to be reduced by approximately half while maintaining full nematocidal efficacy. This reduction in the effective dose is particularly significant, considering the high toxicity of CBF, which has reported LD_50_ values ranging from 2 mg·kg^−1^ in mice to 8 mg·kg^−1^ in rats [62]. In contrast, piperidinium surfactants exhibit significantly lower toxicity, with LD_50_ values ranging from 36.2 to 83.7 mg·kg^−1^ (mice, intraperitoneal administration) [20]. Piperidinium surfactants may enhance the biological activity of CBF and potentially contribute to a reduction in its toxicity to mammals and the environment. The observed synergistic effect is likely due to the surfactants’ ability to alter the permeability of biological membranes, which could enhance the absorption of CBF into nematodes, improving its efficacy at lower concentrations. These results suggest that piperidinium surfactants have the potential to be promising adjuvants in formulations based on CBF, providing a safer and more environmentally friendly approach to nematode management in agriculture.

The ability of piperidinium surfactants to act as adjuvants and enhance the activity of insecticides was also observed in combination with aldicarb. Similar to CBF, aldicarb is a well-known acetylcholinesterase (AChE) inhibitor involved in the regulation of nematode motor activity [70]. Nematode locomotion is a critical acetylcholine (ACh)-mediated behavior and is highly sensitive to substances that influence endogenous ACh levels. The effects of different aldicarb/surfactant combinations on *C. elegans* locomotion are summarized in Table 5. In the control experiments, exposure to aldicarb at 2.5 µM (0.475 µg·mL^−1^) resulted in impaired locomotion in 13% of nematodes after 1 h and approximately threefold higher effects after 2 h. Treatment with surfactants alone did not affect nematode motility. However, in combination with aldicarb, a marked increase in sensitivity to the insecticide was observed, particularly at the longer exposure time (Table 5). The results suggest that *C. elegans* is more responsive to aldicarb when piperidinium surfactants are present.

The observed effects in both the CBF and aldicarb systems can be explained by the ability of piperidinium surfactants to insert into lipid bilayers and cause partial disordering of the lipid packing. This disruption is expected to increase membrane permeability, facilitating the transport of insecticide molecules through biological barriers. Surfactants can therefore be considered as membrane-modifying agents, increasing the accessibility of active compounds to intracellular targets. However, this explanation is indirect and should be treated as a working hypothesis until further evidence is obtained.

## 3. Materials and Methods

### 3.1. Materials

The piperidinium surfactants investigated in this study were synthesized by the quaternization of substituted alkyl piperidines with 1-bromohexadecane in acetonitrile, followed by the recrystallization of the reaction mixture. This process was carried out according to previously published procedures [20,21,71]. The structures of the obtained compounds were confirmed using elemental analysis, IR, NMR ^1^H, and mass spectrometry. These spectroscopic and analytical techniques were consistent with data reported in the literature. Commercially available carbofuran (98%, Sigma-Aldrich, St. Louis, MO, USA), 1,2-dipalmitoyl-*sn*-glycero-3-phosphocholine (DPPC) (≥99%, Sigma-Aldrich, St. Louis, MO, USA), aldicarb (Pestanal^®^ analytical standard, Sigma-Aldrich, St. Louis, MO, USA), as well as reagents for the biochemical experiments were used without further purification. All solutions were prepared using bidistilled water from a Milli-Q system (Millipore SAS, Molsheim, France).

### 3.2. Determination of Nematocidal Activity

Wild-type *C. elegans* strain N2 was obtained from the Caenorhabditis Genetics Center and used as a model organism. Nematodes were cultivated in 10 cm Petri dishes on standard nematode growth medium (NGM), which contained 17 g·L^−1^ Bacto agar, 2.5 g·L^−1^ peptone, 50 mM NaCl, 1 mM MgSO_4_, 1 mM CaCl_2_, 5 mg·L^−1^ cholesterol, and 25 mM potassium phosphate buffer (pH 6.0) and fed with *Escherichia coli* OP50 [36]. All experiments were performed at 22 °C using synchronized young adult worms grown under standard conditions, which were incubated in M9 buffer, containing 3 g·L^−1^ KH_2_PO_4_, 6 g·L^−1^ Na_2_HPO_4_, 5 g·L^−1^ NaCl, and 0.12 g·L^−1^ MgSO_4_. Prior to testing, the nematodes were washed three times with 10 mL of M9 buffer to remove any culture medium, bacteria, or metabolites. Worms were then randomly divided (100 individuals per group) into 10 mL glass centrifuge tubes containing M9 buffer and various concentrations of surfactants, insecticides, or combinations of these compounds. The final volume of the incubation solution was 1 mL. After 24 h, the mortality of the nematodes was assessed using a microscope. Any worms without spontaneous locomotion or unresponsive to gentle stimulation were counted as dead.

The effect of piperidinium surfactants and aldicarb on *C. elegans* swimming behavior induced by mechanical stimulation was evaluated using individually incubated nematodes. Worms prepared as described above were transferred individually into 10 mL glass vials containing M9 buffer and the appropriate concentrations of surfactant and/or aldicarb. The final incubation volume was 1 mL. Locomotor activity was assessed visually using an SMZ-05 stereomicroscope (Biobase Group, Jinan, China). Behavioral impairments were defined as a loss of coordinated muscle contractions required for sinusoidal swimming or the inability to sustain swimming for 10 s following stimulation.

All experiments were performed in quadruplicate, and data were processed in accordance with international standards [72,73]. In the toxicity assays, each replicate included 100 nematodes per concentration. In the locomotion assays, 50 nematodes were used per experimental condition. All experimental conditions (temperature, medium composition, developmental stage, and exposure time) were kept constant across all groups. Outcome assessment was performed using standardized and consistently applied scoring criteria at fixed time points.

Photographs of nematodes at different stages of the experiment were taken using an MSHOT MF23 fluorescence microscope (MSHOT, Guangzhou, China) at 10× magnification.

To evaluate the effects of piperidinium surfactants on nematode egg viability, adult worms were treated with sodium hypochlorite until complete lysis. The reaction was stopped by adding M9 buffer, and eggs were collected by centrifugation [74]. For each experimental condition, eggs were collected from synchronized *C. elegans* populations, using an equal number of hermaphrodites from a single culture, to ensure consistency in the starting biological material. The isolated eggs were incubated for 2 h in surfactant solutions of varying concentrations, washed with M9 buffer, and transferred onto Petri dishes containing NGM agar seeded with *E. coli* OP50. Equal volumes of egg suspensions were transferred to each plate to ensure comparable initial conditions. Culture development was monitored for 10 days. Egg development experiments were conducted using four separate cultures for each surfactant, and all experiments were repeated four times independently.

### 3.3. Statistical Analysis

All nematode experiments were performed in quadruplicate (n = 4), and the results are presented as mean ± SEM. Statistical significance was determined using one-way or two-way analysis of variance (ANOVA) with a Tukey honestly significant difference post hoc test. Differences were considered statistically significant at *p* < 0.05. All statistical analyses were conducted using OriginLab Pro 2021 software (OriginLab Corporation, Northampton, MA, USA).

### 3.4. Turbidimetric Measurements

DPPC-based liposomes were prepared using the thin-film hydration method, as described in Ref. [20]. A lipid film was formed and hydrated in water at 60 °C with vigorous stirring. The resulting suspension underwent three freeze–thaw cycles and was then extruded through a LiposoFast Basic extruder (Avestin, Ottawa, ON, Canada) using polycarbonate membranes with a pore diameter of 100 nm.

Turbidimetric measurements were carried out using a Specord 250 PLUS spectrophotometer (Analytik Jena AG, Jena, Germany). Primary turbidimetric curves were analyzed using the Van’t Hoff two-state model. The inflection point of the curve corresponds to the main phase transition of DPPC from the gel to the liquid-crystalline phase, as described in Ref. [52].

### 3.5. Dynamic and Electrophoretic Light Scattering

The hydrodynamic diameter (D_h_) and zeta potential (ZP) of the liposomal particles were measured using a Zetasizer Nanoparticle size analyzer (Malvern Instruments Ltd., Malvern, UK). A He-Ne laser with a wavelength of 633 nm and a power of 10 mW was used as a light source. The backscattering angle was set to 173°, and the acquisition time was 5–8 min. Data were analyzed using a multichannel correlator connected to an IBM-compatible PC with specialized software to evaluate the effective D_h_ and ZP of the particles. Dynamic and electrophoretic light scattering measurements were carried out at 25 °C.

## 4. Conclusions

In this study, the toxic effect of a series of hexadecylpiperidinium surfactants containing alkyl, benzyl, and hydroxyl substituents was systematically investigated against *C. elegans* (wild-type strain N2) as a model organism. It was shown that increasing the hydrophobicity of the substituent in the piperidinium head group led to a decrease in nematode mortality. The most active compound exhibited markedly higher activity than the commercial insecticide CBF, and at a concentration of 50 μg·mL^−1^, was capable of inducing nearly complete nematode mortality within 24 h, effectively suppressing culture development. In addition to their activity against adult nematodes, piperidinium surfactants were also found to inhibit the development of early life stages. Experiments with nematode eggs obtained by lysis showed that at 100 μg·mL^−1^, all tested compounds suppressed culture development, while PMe-16 remained effective at concentrations as low as 25 μg·mL^−1^. When piperidinium surfactants were used as adjuvants in insecticidal formulations containing CBF or aldicarb, a significant synergistic effect was observed. The addition of the most effective surfactants (PMe-16 and 4-HPMe-16) at a concentration of 6.25 μg·mL^−1^ enabled a nearly twofold reduction in the dose of CBF while maintaining full nematocidal activity. These results indicate that piperidinium surfactants may be promising adjuvants for enhancing the effectiveness of conventional nematicides.

## Figures and Tables

**Table 1:** Nematocidal activity of piperidinium surfactants: percentage (%) of nematode mortality after 24 h *.

Compounds	Concentration of Surfactant, μg·mL^−1^				
	100.0	50.0	25.0	12.50	6.25
PMe-16	99.8 ± 0.5 ^a^	99.0 ± 0.8 ^a,b^	82.8 ± 1.4 ^c^	54.0 ± 2.7 ^f^	25.0 ± 4.2 ^g,h,i^
PEt-16	99.5 ± 0.6 ^a,b^	95.8 ± 2.2 ^a,b^	68.5 ± 2.1 ^d^	27.0 ± 1.8 ^g,h^	13.0 ± 2.4 ^l,m^
PBu-16	99.3 ± 1.5 ^a,b^	79.5 ± 2.9 ^c^	21.0 ± 1.6 ^h^	15.5 ± 2.4 ^j,k,l^	>5.5 ^n,o^
3-HPMe-16	81.0 ± 3.6 ^c^	80.0 ± 2.9 ^c^	64.5 ± 3.4 ^d,e^	19.7 ± 2.2 ^j,k^	>7.0 ^m,n,o^
4-HPMe-16	94.0 ± 1.8 ^a,b^	93.0 ± 2.4 ^b^	58.0 ± 2.6 ^e,f^	29.0 ± 3.6 ^g^	14.0 ± 1.8 ^k,l^
Benz-16	30.0 ± 4.2 ^†^	12.0 ± 2.9 ^†^	>5.0 ^†^	–	–
1-Benz-3-HP-16	23.0 ± 3.9 ^†^	18.0 ± 3.1 ^†^	12.3 ± 2.2 ^†^	–	–
1-Benz-4-HP-16	31.0 ± 2.9 ^†^	10.0 ± 1.8 ^†^	>5.0 ^†^	–	–
CBF	67.0 ± 3.6 ^d^	26.5 ± 1.9 ^g,h^	9.0 ± 0.8 ^l,m,n^	1.5 ± 0.6 ^o^	>0.5 ^o^

* Data are presented as the mean ± standard error of the mean (SEM) (n = 4). Statistical analysis was performed using a two-way analysis of variance (two-way ANOVA) followed by a Tukey’s post hoc test using OriginLab Pro 2021 software. Statistically significant differences between groups are indicated by different letters (a–o) at *p* < 0.05; identical letters denote no significant difference (Figure S2). Piperidinium surfactants containing a benzyl fragment (Benz-16, 1-Benz-3-HP-16, and 1-Benz-4-HP-16) were analyzed separately at each concentration level using one-way ANOVA with a Tukey test (Figure S3). The “†” symbol indicates statistically significant differences for this subgroup compared to other alkyl- and hydroxyl-substituted piperidinium surfactants as determined by a one-way ANOVA (*p* < 0.0001). The “–” symbol indicates that no value was determined due to the absence of detectable activity (mortality < 5%).

**Table 2:** Effects of piperidinium surfactants on the viability and development of nematode eggs obtained by lysis over 10 days.

Surfactant	Concentration of Surfactant, μg·mL^−1^		
	100.0	50.0	25.0
PMe-16	No culture development		
PEt-16	No culture development	Normal culture development	
4-HPMe-16	No culture development		
3-HPMe-16	No culture development		

**Table 3:** Molar ratios of surfactants-to-lipids and concentrations causing bilayer loosening or disruption, and the DLS data of the systems.

System	Molar Ratio of Components	T_m_, °C	C_Surf_, mM	C_Surf_, µg·mL^−1^	D_h_, nm *	PdI	ZP, mV *
DPPC	–	42.60	–	–	106 ± 1	0.050 ± 0.006	4.1 ± 0.2
DPPC:PMe-16 **	1:3	42.05	0.23	93.04	–	–	–
	1:1	39.52	0.70	283.16	–	–	–
DPPC:PEt-16	1:5	40.80	0.14	58.60	–	–	–
	1:1	39.67	0.70	292.98	26 ± 1	0.525 ± 0.028	45.0 ± 2.0
DPPC:PBu-16	1:5	39.07	0.14	62.52	–	–	–
	1:3	38.50	0.23	102.72	173 ± 1	0.152 ± 0.020	47.5 ± 0.4
DPPC:4-HPMe-16	1:3	42.42	0.23	96.72	–	–	–
	1:1	38.79	0.70	294.36	220 ±13	0.476 ± 0.065	46.4 ± 1.5

* Hydrodynamic diameter and ZP were presented as Z-average values; ** Data were taken from [20].

**Table 4:** The nematocidal activity of formulations with varying concentrations of CBF and piperidinium surfactants: percentage (%) of nematode mortality after 24 h *.

Surfactant	C_Surf_, µg·mL^−1^	C_CBF_, µg·mL^−1^			
		0	100.0	50.0	25.0
None (control)	–	0	67.0 ± 3.6	26.5 ± 1.7	9.0 ± 0.8
PMe-16	6.25	25.0 ± 1.4 ^a^	94.5 ± 1.3 ^a,b,c,d^	69.0 ± 3.5 ^b,c^	78.5 ± 2.4 ^a^
	3.12	12.0 ± 0.8 ^c^	87.0 ± 2.1 ^e,f^	56.5 ± 3.1 ^e^	39.5 ± 3.1 ^c^
	1.56	2.0 ± 0.8 ^e^	78.8 ± 2.5 ^g^	39.5 ± 3.7 ^f^	>8.0 ^e,f^
PEt-16	6.25	13.0 ± 0.8 ^b,c^	93.0 ±1.8 ^b,c,d,e^	88.5 ± 2.4 ^a^	82.5 ± 3.0 ^a^
	3.12	2.0 ± 0.8 ^e^	94.5 ± 3.1 ^a,b,c,d^	66.0 ± 2.6 ^c,d^	13.5 ± 2.6 ^e,f^
	1.56	0	88.5 ± 1.3 ^d,e,f^	42.5 ± 3.0 ^f^	>7.0 ^f^
PBu-16	6.25	5.5 ± 0.6 ^d^	94.0 ± 2.6 ^a,b,c,d^	74.0± 3.9 ^b^	29.0 ± 2.4 ^d^
	3.12	3.0 ± 0.8 ^e^	90.3 ± 2.6 ^c,d,e,f^	46.5 ± 3.5 ^f^	29.0 ± 4.1 ^d^
	1.56	0	86.0 ± 4.5 ^f^	42.0 ± 2.9 ^f^	15.0 ± 2.1 ^e^
3-HPMe-16	6.25	7.0 ± 0.8 ^d^	99.5 ± 0.6 ^a^	70.5 ± 1.9 ^b,c^	27.5 ± 2.1 ^d^
	3.12	1.8 ± 0.9 ^e^	86.5 ± 3.9 ^f^	60.5 ± 3.3 ^d,e^	30.5 ± 2.1 ^d^
	1.56	0	63.8 ± 3.5 ^h^	31.5 ± 2.5 ^g^	>7.5 ^f^
4-HPMe-16	6.25	14.0 ± 0.8 ^b^	95.0 ± 1.8 ^a,b,c^	70.5 ± 3.3 ^b,c^	49.0 ± 2.4 ^b^
	3.12	6.0 ± 0.8 ^d^	97.5 ± 0.6 ^a,b^	75.0 ± 2.9 ^b^	44.5 ± 3.1 ^b,c^
	1.56	0	87.5 ± 2.3 ^e,f^	68.0 ± 3.7 ^b,c,d^	25.0 ± 21 ^d^

* Data are presented as the mean ± SEM (n = 4). Statistical analysis was conducted using two-way analysis of variance (two-way ANOVA), followed by Tukey’s post hoc test. Statistically significant differences between groups are denoted by different letters (^a^, ^b^, ^c^, etc.) at *p* < 0.05, while identical letters indicate no significant difference (Figure S13). For comparisons of different CBF concentrations within the same surfactant group, a one-way ANOVA was applied, revealing significant differences in all groups (*p* < 0.05).

**Table 5:** The effect of piperidinium surfactants on the locomotor activity of *C. elegans* in the presence of aldicarb: percentage (%) of nematodes without locomotion disorders.

Surfactant *	Control (Without Nematicide)		Aldicarb **	
	After 1 h	After 2 h	After 1 h	After 2 h
None	100	100	87.5 ± 4.7	65.6 ± 7.2
PMe-16	100	100	81.3 ± 5.6	31.3 ± 4.6
3-HPMe-16	100	100	78.1 ± 5.9	46.9 ± 5.9
4-HPMe-16	100	100	75.0 ± 6.3	31.3 ± 4.6

* The surfactants concentration was 6.25 µg·mL^−1^; ** Aldicarb concentration was 2.5 µM (or 0.475 µg·mL^−1^).

## Data Availability

The data presented in this study are available on request from the corresponding author: Alla Mirgorodskaya.

## Supplementary Materials

The following supporting information can be downloaded at: https://www.mdpi.com/article/10.3390/molecules31091470/s1, Figure S1: EC_50_ values for piperidinium surfactants and CBF; Figure S2: Results of two-way analysis of variance (ANOVA) followed by Tukey’s post hoc multiple comparisons test; Figure S3: One-way ANOVA analysis of nematocidal activity of piperidinium surfactants and CBF at different concentrations; Figure S4: Optical density at 350 nm for liposome suspensions with additives: PEt-16 (A), PBu-16 (B), and 4-HPMe-16 (C); Figure S5: Size distribution of liposomes; Figure S6: Zeta potential of liposomes; Figure S7: Size distribution of liposomes after PEt-16 intercalation; Figure S8: Zeta potential of liposomes after PEt-16 intercalation; Figure S9: Size distribution of liposomes after PBu-16 intercalation; Figure S10: Zeta potential of liposomes after PBu-16 intercalation; Figure S11: Size distribution of liposomes after 4-HPMe-16 intercalation; Figure S12: Zeta potential of liposomes after 4-HPMe-16 intercalation; Figure S13: Two-way ANOVA summary tables (Tukey’s post hoc test) for systems with different CBF concentrations; Figure S14: Nematode mortality in the presence of piperidinium surfactants, CBF (100 µg·mL^−1^), and their combinations (HSA model); Table S1: Experimental and model-calculated nematode mortality for piperidinium surfactants and CBF; Table S2: Synergy analysis of *C. elegans* mortality in combined systems, including Bliss model interaction coefficients and HSA combination index.
