# Supplementary material for: The Use of Piperidinium Surfactants in Nematicide Formulations
Source: Molecules. 2026 Apr 29;31(9):1470. doi: 10.3390/molecules31091470 (PMC13164857; doi:10.3390/molecules31091470)
Supplement: Supplementary file 1 [file molecules-31-01470-s001.zip › molecules-4232453-supplementary.pdf]

The use of piperidinium surfactants in nematicide formulations

Rushana Kushnazarova [1], Alla Mirgorodskaya [1,*], Eugeny Nikitin [1], Anastasia Egorova [2], Alsu Gatiyatullina [2], Tatiana Kalinnikova [2], Lucia Zakharova [1]

[1] Arbuzov Institute of Organic and Physical Chemistry, FRC Kazan Scientific Center of RAS, Arbuzov Str. 8, 420088 Kazan, Russia

[2] Research Institute for Problems of Ecology and Mineral Wealth Use of Tatarstan Academy of Sciences, Daurskaya Str. 28, 420087 Kazan, Russia

* Correspondence: mirgoralla@mail.ru

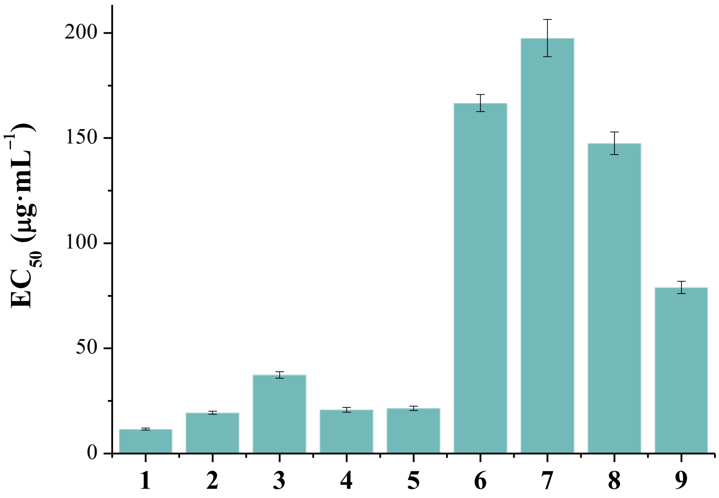

Figure S1. EC_{50} values for piperidinium surfactants and carbofuran (CBF): **1** – PMe-16, **2** – PEt-16, **3** – PBu-16, **4** – 3-HPMe-16, **5** – 4-HPMe-16, **6** – Benz-16, **7** – 1-Benz-3-HP-16, **8** – 1-Benz-4-HP-16, **9** – CBF.

A

Interactions 's

Compounds	Conce	Mean	Groups														
PMe-16	100	99.75	A														
PEt-16	100	99.5	A	B													
PBu-16	100	99.25	A	B													
PMe-16	50	99	A	B													
PEt-16	50	95.75	A	B													
4-HPMe-16	100	94	A	B													
4-HPMe-16	50	93		B													
PMe-16	25	83			C												
3-HPMe-16	100	81			C												
3-HPMe-16	50	80			C												
PBu-16	50	79.5			C												
PEt-16	25	68.5				D											
CBF	100	67				D											
3-HPMe-16	25	64.5				D	E										
4-HPMe-16	25	58					E	F									
PMe-16	12.5	54						F									
4-HPMe-16	12.5	29							G								
PEt-16	12.5	27							G	H							
CBF	50	26.5							G	H							
PMe-16	6.25	25							G	H	I						
PBu-16	25	21								H	I	J					
3-HPMe-16	12.5	19.75									I	J	K				
PBu-16	12.5	15.5										J	K	L			
4-HPMe-16	6.25	14											K	L			
PEt-16	6.25	13												L	M		
CBF	25	9												L	M	N	
3-HPMe-16	6.25	6.75													M	N	O
PBu-16	6.25	4.75														N	O
CBF	12.5	1.5															O
CBF	6.25	0.5															O

Means that do not share a letter are significantly different.

B

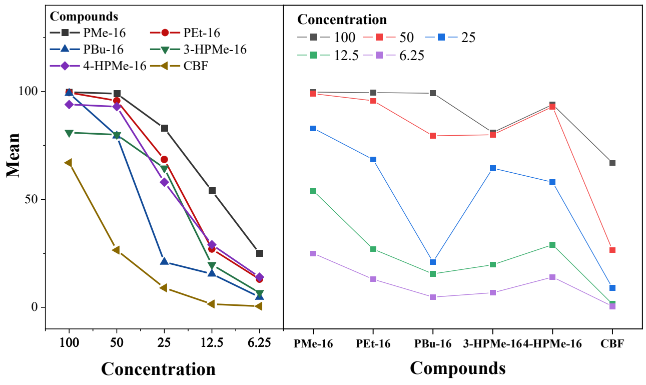

Figure S2. Results of two-way analysis of variance (ANOVA) followed by Tukey's post hoc multiple comparisons test (OriginPro software). Different superscript letters (**A-O**) indicate statistically significant differences among groups (p < 0.05), whereas groups sharing at least one letter are not significantly different. (**A**) ANOVA summary table; (**B**) interaction plot.

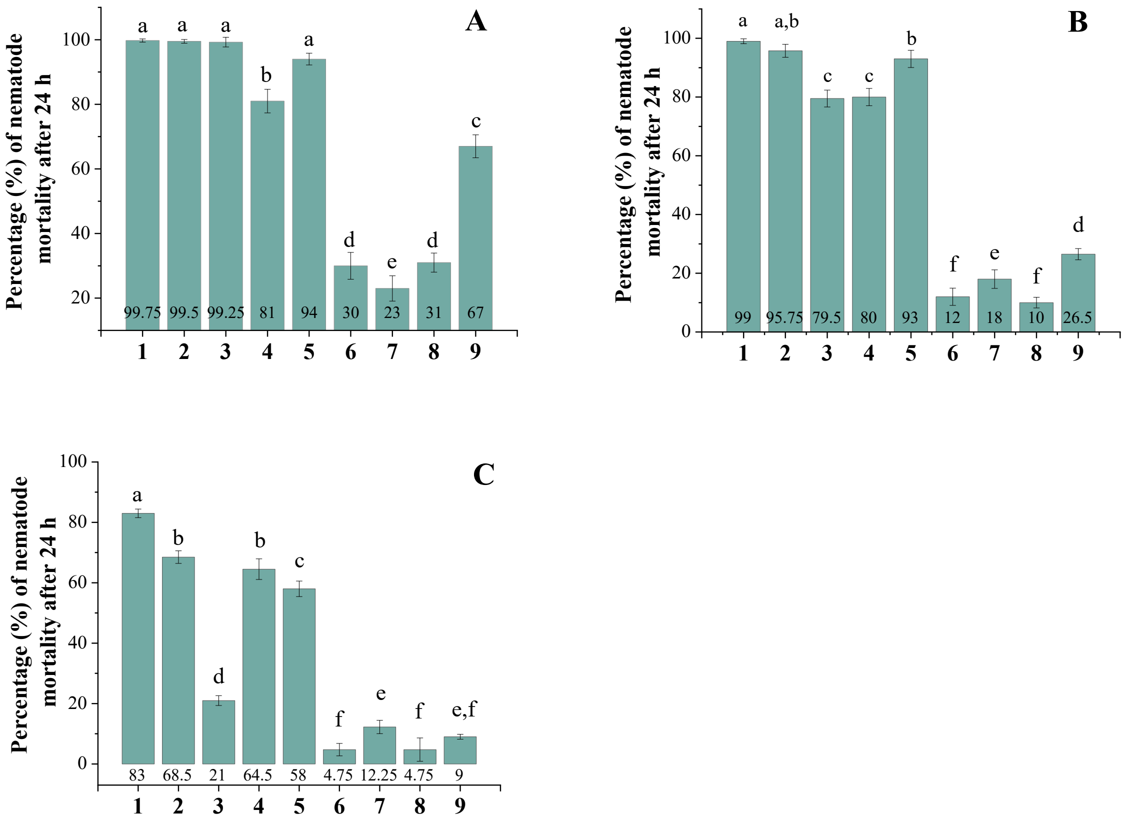

Figure S3. One-way ANOVA mean plots of nematocidal activity of piperidinium surfactants (**1** – PMe-16, **2** – PEt-16, **3** – PBu-16, **4** – 3-HPMe-16, **5** – 4-HPMe-16, **6** – Benz-16, **7** – 1-Benz-3-HP-16, **8** – 1-Benz-4-HP-16) and carbofuran (**9**) at different concentration: (**A**) 100 μg·mL–1; (**B**) 50 μg·mL^{-1}; (**C**) 25 μg·mL^{-1}.s. Different letters indicate statistically significant differences among groups based on Tukey's post hoc test (p < 0.05), whereas groups sharing at least one letter are not significantly different. Data are presented as mean ± SEM (n = 4).

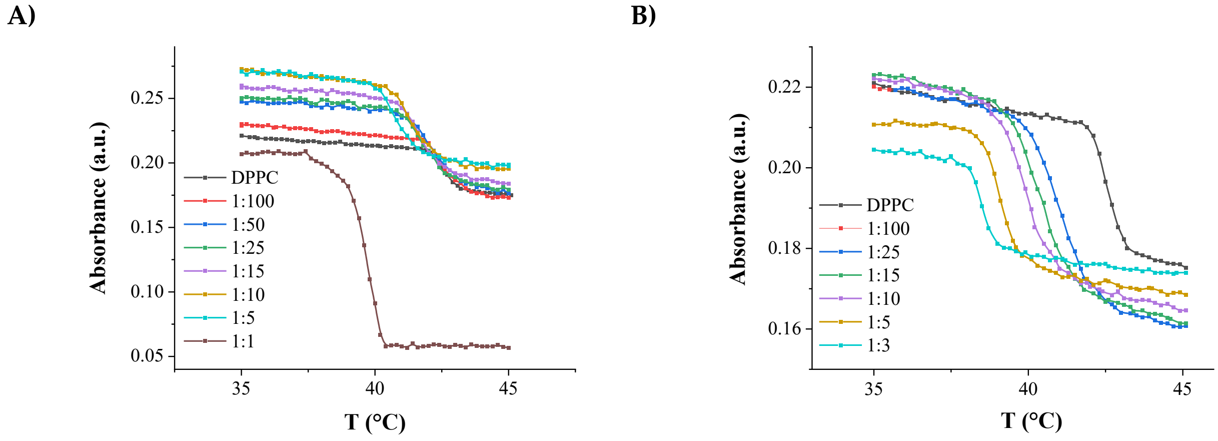

C)

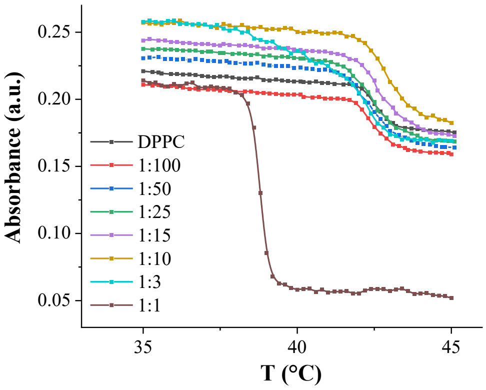

Figure S4. Initial curves of changes in optical density at a wavelength of 350 nm for samples of a suspension of DPPC-based liposomes under conditions of varying additives: PEt-16 (**A**), PBu-16 (**B**), and 4-HPMe-16 (**C**).

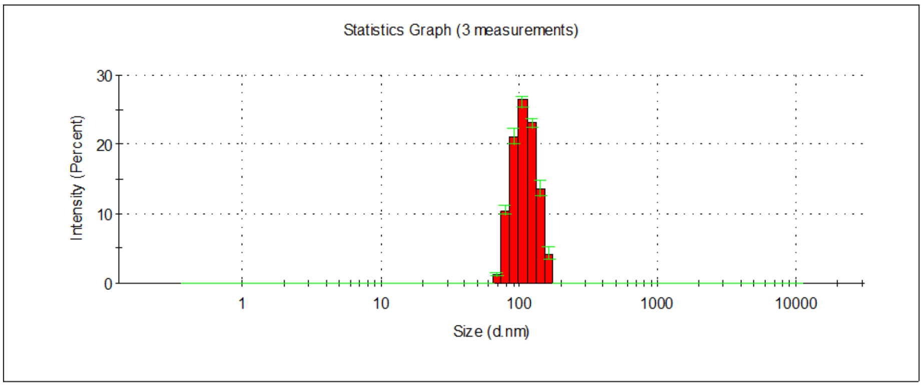

Figure S5. The hydrodynamic diameter of DPPC-based liposomes (0.7 mM), averaged by intensity; 25 °C.

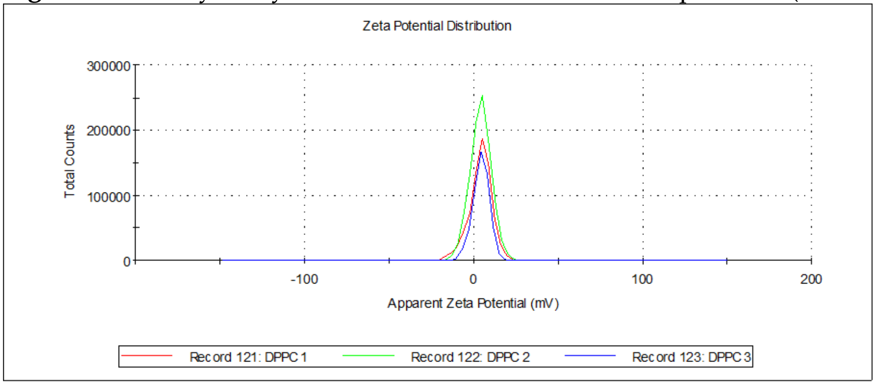

Figure S6. Zeta potential of DPPC-based liposomes (C = 0.7 mM); 25 °C.

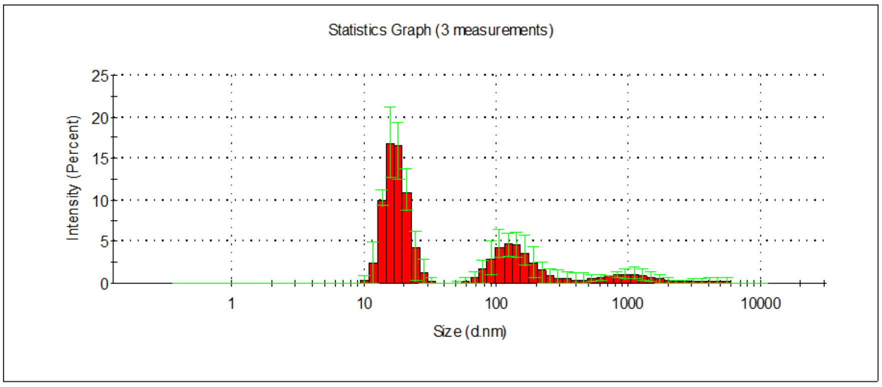

Figure S7. The hydrodynamic diameter of DPPC-based liposomes after the intercalation of PEt-16, averaged by intensity. The molar ratio of PEt-16 to DPPC is 1:1; 25 °C.

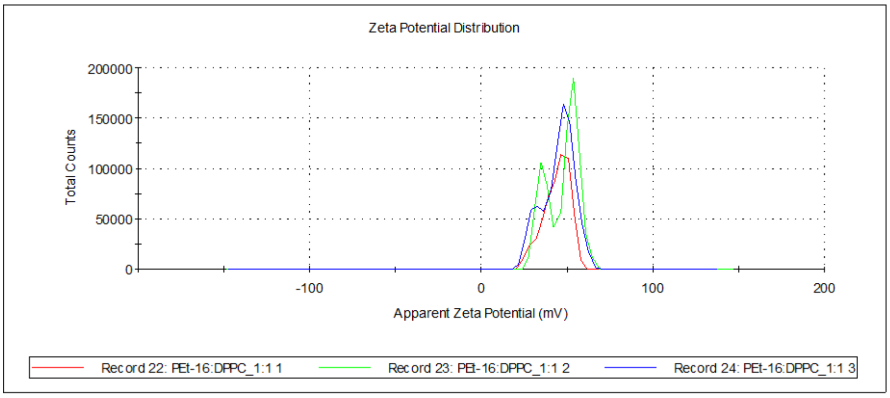

Figure S8. Zeta potential of the system after the intercalation of PEt-16. The molar ratio of PEt-16 to DPPC is 1:1; 25 °C.

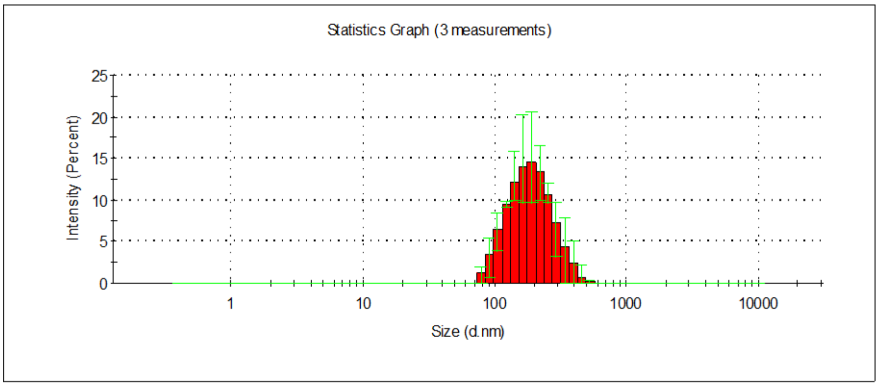

Figure S9. The hydrodynamic diameter of DPPC-based liposomes after the intercalation of PBu-16, averaged by intensity. The molar ratio of PBu-16 to DPPC is 1:3; 25 °C.

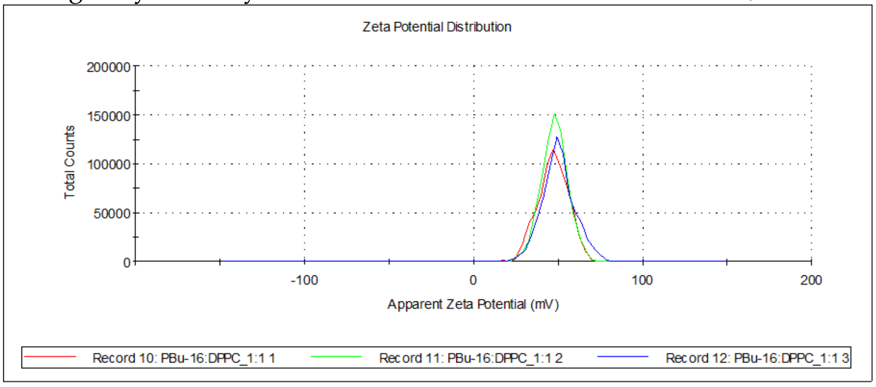

Figure S10. Zeta potential of the system after the intercalation of PBu-16. The molar ratio of PBu-16 to DPPC is 1:3; 25 °C.

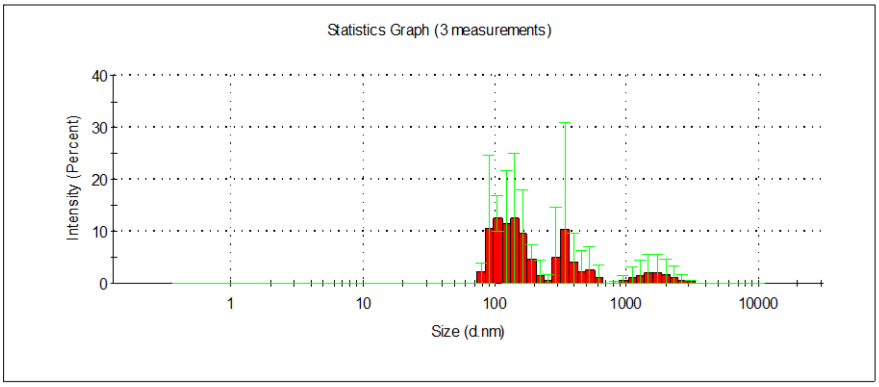

Figure S11. The hydrodynamic diameter of DPPC-based liposomes after the intercalation of 4-HPMe-16, averaged by intensity. The molar ratio of 4-HPMe-16 to DPPC is 1:1; 25 °C.

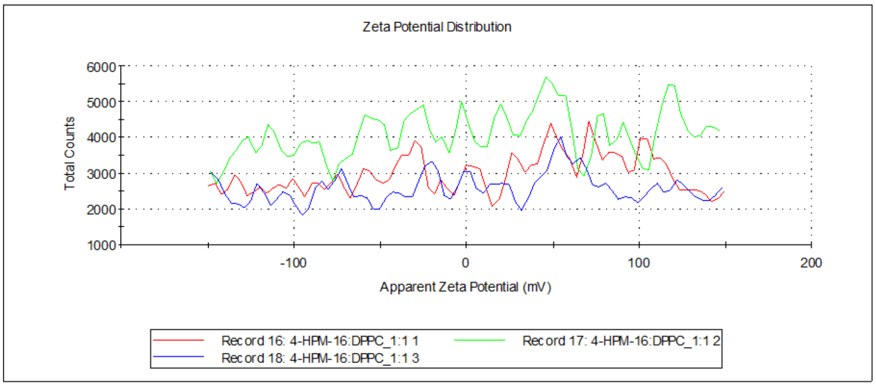

Figure S12. Zeta potential of the system after the intercalation of 4-HPMe-16. The molar ratio of 4-HPMe-16 to DPPC is 1:1; 25 °C.

Interactions 's

Surfactant	C(surf)	Mean	Groups					
PMe-16	6.25	24	A					
4-HPMe-16	6.25	14		B				
PEt-16	6.25	13		B	C			
PMe-16	3.12	12			C			
3-HPMe-16	6.25	7				D		
4-HPMe-16	3.12	6				D		
PBu-16	6.25	5.5				D		
PBu-16	3.12	3					E	
PEt-16	3.12	2					E	
PMe-16	1.56	2					E	
3-HPMe-16	3.12	1.75					E	F
PBu-16	1.56	0						F
3-HPMe-16	1.56	0						F
PEt-16	1.56	0						F
4-HPMe-16	1.56	0						F

Means that do not share a letter are significantly different.

A

Interactions 's

Surfactant	C(surf)	Mean	Groups							
3-HPMe-16	6.25	99.5	A							
4-HPMe-16	3.12	97.5	A	B						
4-HPMe-16	6.25	95	A	B	C					
PMe-16	6.25	94.5	A	B	C	D				
PEt-16	3.12	94.5	A	B	C	D				
PBu-16	6.25	94	A	B	C	D				
PEt-16	6.25	93		B	C	D	E			
PBu-16	3.12	90.25			C	D	E	F		
PEt-16	1.56	88.5				D	E	F		
4-HPMe-16	1.56	87.5					E	F		
PMe-16	3.12	87					E	F		
3-HPMe-16	3.12	86.5						F		
PBu-16	1.56	86						F		
PMe-16	1.56	78.75							G	
3-HPMe-16	1.56	63.75								H

Means that do not share a letter are significantly different.

B

Interactions 's

Surfactant	C(surf)	Mean	Groups						
PEt-16	6.25	88.5	A						
4-HPMe-16	3.12	75		B					
PBu-16	6.25	74		B					
4-HPMe-16	6.25	70.5		B	C				
3-HPMe-16	6.25	70.5		B	C				
PMe-16	6.25	69		B	C				
4-HPMe-16	1.56	68		B	C	D			
PEt-16	3.12	66			C	D			
3-HPMe-16	3.12	60.5				D	E		
PMe-16	3.12	56.5					E		
PBu-16	3.12	46.5						F	
PEt-16	1.56	42.5						F	
PBu-16	1.56	42						F	
PMe-16	1.56	39.5						F	
3-HPMe-16	1.56	31.5							G

Means that do not share a letter are significantly different.

C

Interactions 's

Surfactant	C(surf)	Mean	Groups					
PEt-16	6.25	82.5	A					
PMe-16	6.25	78.5	A					
4-HPMe-16	6.25	49		B				
4-HPMe-16	3.12	44.5		B	C			
PMe-16	3.12	39.5			C			
3-HPMe-16	3.12	30.5				D		
PBu-16	6.25	29				D		
PBu-16	3.12	29				D		
3-HPMe-16	6.25	27.5				D		
4-HPMe-16	1.56	25				D		
PBu-16	1.56	15					E	
PEt-16	3.12	13.5					E	F
PMe-16	1.56	8					E	F
3-HPMe-16	1.56	7.25						F
PEt-16	1.56	6.75						F

Means that do not share a letter are significantly different.

D

Figure S13. Two-way ANOVA summary tables (Tukey's post hoc test). Different superscript letters (A-H) indicate significant differences among groups within each panel ($p < 0.05$). Groups sharing at least one letter are not significantly different. Analyses were performed at fixed concentrations of carbofuran (CBF): (**A**) 0 μg·mL^{-1}, (**B**) 100 μg·mL^{-1}, (**C**) 50 μg·mL^{-1}, (**D**) 25 μg·mL^{-1}.

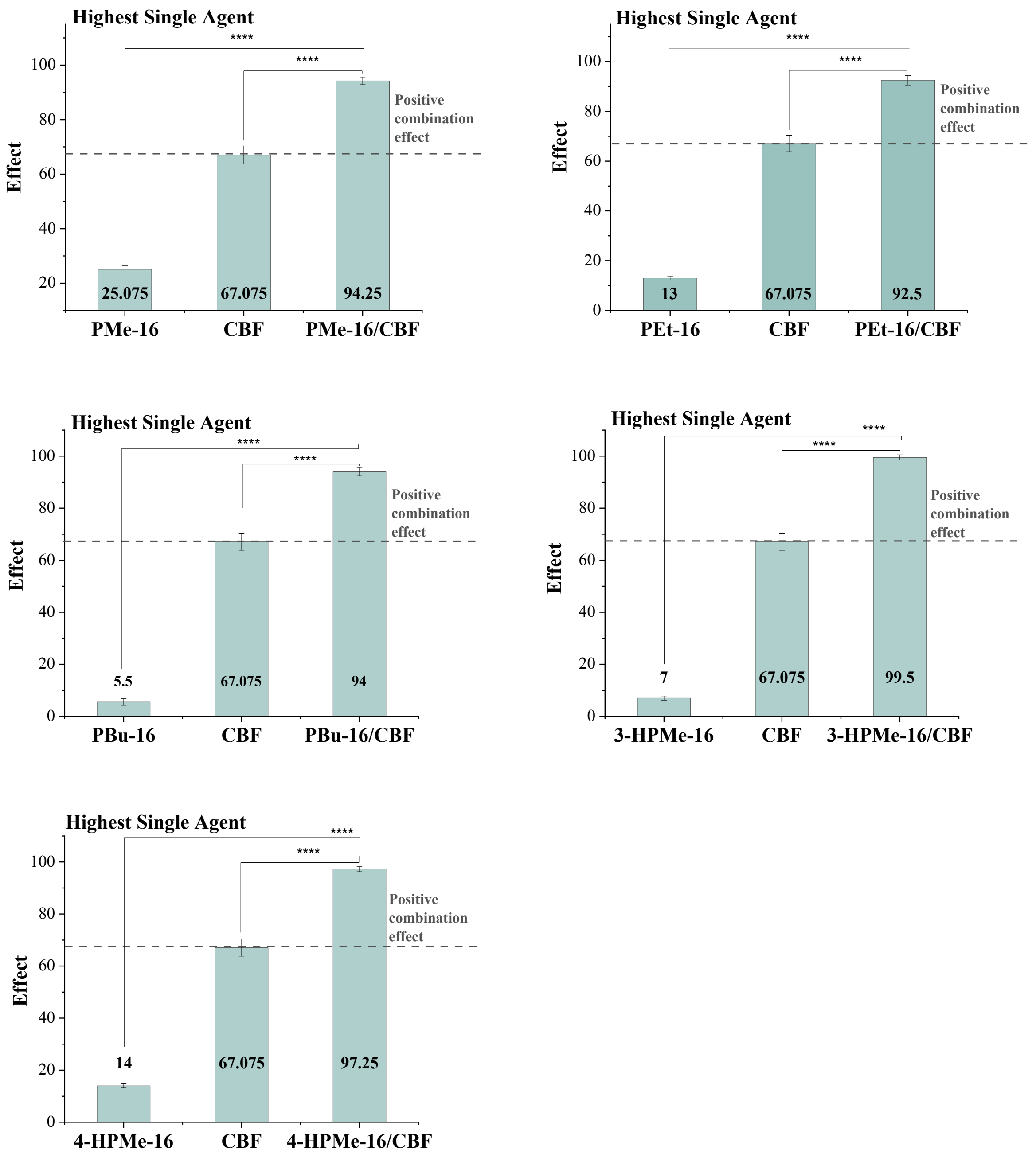

Figure S14. Nematode mortality in the presence of piperidinium surfactants (6.25 μg·mL^{-1}), carbofuran (100 μg·mL^{-1}), and their combinations (HSA model). Statistical significance relative to individual components was determined using one-way ANOVA followed by Tukey's test (**** $p < 0.0001$).

Table S1. Experimental and additive model-calculated values of nematode mortality at varying concentrations of piperidinium surfactants and carbofuran (CBF).

Surfactant	C_{Surf}, μg·mL^{-1}	C_{CBF}, μg·mL^{-1}				C_{CBF}, μg·mL^{-1}		
		0	100	50	25	100	50	25
None (control)	–	0	67	26.5	9	Additive values		
PMe-16	6.25	25.0	94.3	69.0	78.5	75.25	44.88	31.75
	3.12	12.0	87.0	56.5	39.5	70.96	35.32	19.92
	1.56	2.0	78.5	39.5	8.0	67.66	27.97	10.82
PEt-16	6.25	13.0	92.5	88.5	82.5	71.29	36.06	20.83

	3.12	2.0	94.5	66.0	13.5	67.66	27.97	10.82
	1.56	0	88.5	42.5	7.0	67.00	26.50	9.00
PBu-16	6.25	5.5	94.0	74.0	29.0	68.82	30.54	14.01
	3.12	3.0	90.3	46.5	29.0	67.99	28.71	11.73
	1.56	0	86.0	42.0	15.0	67.00	26.50	9.00
3-HPMe-16	6.25	7.0	100.0	70.5	27.5	69.31	31.65	15.37
	3.12	1.6	86.5	60.5	30.5	67.53	27.68	10.46
	1.56	0	63.0	31.5	7.5	67.00	26.50	9.0
4-HPMe-16	6.25	14.0	95.0	70.5	49.0	71.62	36.79	21.74
	3.12	6.0	97.5	75.0	44.5	68.98	30.91	14.46
	1.56	0	87.5	68.0	25.0	67.00	26.50	9.00

Table S2. Comparison of *C. elegans* mortality and synergy evaluation with the combined use of piperidinium surfactants and carbofuran: experimental data, Bliss model interaction coefficient, and combination index (CI) according to the Highest Single Agent (HSA) model.

**Surfactant**	**C_{Surf}, μg·mL^{-1}**	**C_{CBF}, μg·mL^{-1}**				**C_{CBF}, μg·mL^{-1}**			**C_{CBF}, μg·mL^{-1}**		
		0	100	50	25	100	50	25	100	50	25
None (control)	–	0	67.1	26.5	9.0	Interaction coefficient (Bliss)			Combination index (HSA)		
PMe-16	6.25	25.0	94.3	69.0	78.5	1.25	1.54	2.47	0.71	0.38	0.32
	3.12	12.0	87.0	56.5	39.5	1.23	1.60	1.98	0.77	0.47	0.30
	1.56	2.0	78.5	39.5	8.0	1.16	1.41	0.74	0.85	0.67	>1.0
PEt-16	6.25	13.0	92.5	88.5	82.5	1.30	2.45	3.96	0.73	0.30	0.11
	3.12	2.0	94.5	66.0	13.5	1.40	2.36	1.25	0.71	0.40	0.67
	1.56	0	88.5	42.5	7.0	1.32	1.60	0.78	0.76	0.62	1.28
PBu-16	6.25	5.5	94.0	74.0	29.0	1.37	2.42	2.07	0.71	0.36	0.31
	3.12	3.0	90.3	46.5	29.0	1.33	1.62	2.47	0.74	0.57	0.31
	1.56	0	86.0	42.0	15.0	1.28	1.58	1.67	0.78	0.63	0.60
3-HPMe-16	6.25	7.0	99.8	70.5	27.5	1.44	2.23	1.79	0.67	0.37	0.33
	3.12	1.6	86.5	60.5	30.5	1.28	2.19	2.92	0.77	0.44	0.30
	1.56	0	63.0	31.5	7.5	0.94	1.19	0.83	1.06	0.84	1.2
4-HPMe-16	6.25	14.0	95.0	70.5	49.0	1.33	1.92	2.25	0.71	0.37	0.18
	3.12	6.0	97.5	75.0	44.5	1.41	2.43	3.08	0.69	0.35	0.20
	1.56	0	87.5	68.0	25.0	1.31	2.57	2.78	0.77	0.39	0.36
